# Self-Directed Interventions to Promote Weight Loss: A Systematic Review of Reviews

**DOI:** 10.2196/jmir.2857

**Published:** 2014-02-19

**Authors:** Jason Tang, Charles Abraham, Colin Greaves, Tom Yates

**Affiliations:** ^1^University of Exeter Medical SchoolUniversity of ExeterExeterUnited Kingdom; ^2^NIHR Leicester-Loughborough Diet, Lifestyle, and Physical Activity Biomedical Research UnitLeicesterUnited Kingdom; ^3^Diabetes Research CentreCollege of Medicine, Biological Sciences and PsychologyUniversity of LeicesterLeicesterUnited Kingdom

**Keywords:** weight loss, obesity, Internet, eHealth, home-based, text message, self-delivered, intervention, systematic review

## Abstract

**Background:**

A wide range of self-directed weight-loss interventions are available, providing users with a variety of tools delivered through various formats to regulate weight-related behavior patterns. However, it is unclear how effective self-directed interventions are and how they promote weight loss and weight maintenance.

**Objective:**

A systematic review of reviews was conducted to examine the effectiveness of such interventions and to identify intervention content associated with effectiveness.

**Methods:**

MEDLINE, Embase, PsycINFO, CINAHL, and the Cochrane Library for systematic reviews were searched from 2000-2012 for reviews of the effectiveness of self-directed interventions on weight loss and weight maintenance in adults. Two reviewers used predefined inclusion criteria to select relevant reviews and assess their quality using the Overview Quality Assessment Questionnaire (OQAQ). We extracted data on effectiveness and on relationships between intervention characteristics and effectiveness.

**Results:**

Twenty reviews were included and quality assessed. Findings relevant to self-directed interventions, including interactive websites, smartphone applications, and text messaging (short message service, SMS) were summarized. Findings were mixed but promising. For example, one review of Internet-based interventions found that, when used in conjunction with standard weight loss programs, these interventions resulted in a significant average increase in weight loss of 1.5 kg over evaluation periods. Unfortunately, only 7 of 20 reviews were of high methodological quality according to OQAQ scores, and only 4 employed meta-analyses. Few reviews linked intervention content to effectiveness.

**Conclusions:**

Current evidence suggests that self-directed interventions can independently promote weight loss and can augment interventions involving personal contact. Particular change techniques and delivery modes including individualized feedback, email counseling, and online social support appear to enhance effectiveness. Further reviews of the content of self-directed weight-loss intervention studies are needed to clarify which change techniques delivered through which delivery formats optimize intervention effectiveness.

## Introduction

Weight reduction is a global health priority because being overweight or obese is associated with multiple health problems, including the leading causes of preventable death such as cardiovascular disease, type 2 diabetes, and particular cancers [1,2]. Yet the prevalence of obesity and health services resources devoted to treating its consequences are increasing internationally. In the United States, for example, 68% of adults are overweight or obese accounting for more than 20% of health care costs [3].

Pharmaceutical and bariatric surgery treatments are effective for some overweight and obese people but are expensive and often accompanied by adverse side effects. Consequently, they tend to be weight loss treatments of last resort [4]. Reversing population obesity trends depends on prompting widespread changes in diet and physical activity patterns [5,6]. Promotion of initiation and maintenance of weight changes will be optimized by an understanding of the processes regulating eating and physical activity patterns [7,8].

Effective face-to-face interventions have been developed to promote weight loss through changes in diet and physical activity [9], but these require substantial, specialist delivery personnel and resources [10]. Consequently, more intensive (higher contact frequency) and expensive interventions are most effective. Further research is warranted on intervention formats that could reduce costs without decreasing effectiveness [11]. Effective, high-intensity, low-cost interventions may be developed if participants self-deliver intervention content using printed media (eg, self-help manuals) or interactive software (on mobile phones, the Internet, or other online mobile devices). We use the term “self-directed interventions” to mean those that require minimal professional contact (for example, provision of initial instructions) or no professional contact and can be easily used with existing infrastructure and in the context of users’ everyday lives. Many such interventions have been developed [12], and although attrition rates are often high [13], such interventions have been found to be effective for a broad range of health behavior changes including improving diabetes self-management and smoking cessation [14,15].

Self-directed interventions are likely to be most effective when they empower participants to control and regulate their own thoughts, feelings, and behaviors, thereby changing psychological and environmental prompts to weight-gain behaviors [8]. These interventions are not only self-directed but also “self-regulatory” in that people are taught to change the regulatory processes that maintain current behavior patterns and establish new ones. For example, it has been suggested that prompting self-regulation through self-monitoring of behavior, providing timely feedback on behavior changes, prompting goal setting, and specific action planning are all associated with effectiveness in dietary and physical activity interventions [16,11]. Nonetheless, it remains unclear which self-directed weight loss interventions are effective and why. For example, which combinations of behavior change techniques [17,18] targeting which behavior regulation processes delivered through which particular delivery formats [19] optimize weight loss and weight maintenance over time?

Considerable research has been devoted to developing and evaluating self-directed, weight loss interventions, and a number of recent reviews are available. Some reviews have focused solely on studies evaluating interventions using weight loss outcomes [20], while others have included studies evaluating interventions in terms of weight loss alongside studies using other outcome measures such as self-report behavioral measures.

In a systematic review of reviews, Kohl and Crutzen examined the efficacy, use, and reach of Internet-based interventions for lifestyle changes in physical activity, dietary behaviors, smoking, alcohol consumption, and condom use [21]. One meta-analysis included in this review found that Internet-based interventions of longer duration, based on social cognitive theories, and including educational components with regular updates of intervention content increased physical activity levels [22]. These reviewers also reported that interactive elements, such as chat rooms and online peer support, were associated with greater efficacy. However, identification of such components across interventions was rare.

We are not aware of any previous review of reviews of self-directed interventions evaluated in terms of weight loss outcomes. We therefore conducted a systematic review of reviews to summarize efficacy evidence and design features of self-directed interventions designed to reduce weight and sustain weight maintenance. Within identified reviews, we focused on the conclusions that reviewers drew about interventions evaluated in terms of weight loss. This meant that, for some reviews, all the included primary studies were relevant to our research questions, while for others, a minority of the primary studies were relevant.

Our review aimed to summarize evidence in relation to three key questions:

1. How effective are self-directed weight loss interventions?
2. Is effectiveness enhanced by use of particular change techniques?
3. Is effectiveness enhanced by using particular delivery formats?

## Methods

### Review Inclusion Criteria

To meet these aims, we included reviews based on systematic literature searches published in English between 2000 and 2012 that included at least one primary intervention evaluation:

1. Of an individual-level, self-directed weight loss intervention targeting healthy adults (18 years or over) who were normal weight, sedentary, overweight, or obese. Normal weight intervention participants were included because such studies are important to understanding what works best in prevention of weight gain and maintenance of normal weight in nonclinical populations.
2. Targeting physical activity, diet, or both and were evaluated using at least one weight-related outcome (eg, weight, body mass index [BMI], waist circumference, waist to hip ratio).
3. Employed randomized controlled trials (RCTs), observational, quasi-experimental, and/or cohort studies. Comparison groups could include usual care, other interventions, or no intervention.

### Search Strategy

Reviews that met these inclusion criteria were searched for on the bibliographic electronic databases MEDLINE (Ovid), Embase (Ovid), PsycINFO (Ovid), CINAHL, and the Cochrane Library. Full searches applied in each database are available from the authors.

### Study Selection

The first author examined the titles and abstracts of articles identified by our search against the predefined inclusion criteria. A second researcher repeated this process, and discrepancies were resolved through discussion. Full text articles were obtained and assessed to ensure correspondence to inclusion criteria by the first 3 authors. Disagreements were resolved through discussion, and reasons for exclusion were outlined for each review. See Multimedia Appendix 1 for a list of included and excluded reviews (n=32). References in eligible reviews were checked to identify further relevant reviews.

### Quality Assessment

The quality of each full-text article that met the inclusion criteria was rated by the first and second authors using the Overview Quality Assessment Questionnaire (OQAQ) [23]. Each review was scored against a checklist of nine standard items, including transparency, selection bias, study quality, and replicability. The few scoring disagreements arising were resolved through discussion. Following Greaves et al’s review of reviews on components associated with effectiveness in dietary and physical activity intervention evaluations [11], we labeled reviews as high quality if they scored 14-18 on the OQAQ. Those falling slightly below this threshold (11-13) were labeled medium quality 11-13, and reviews scoring below 11 were regarded as low quality.

### Data Extraction

From each included review, we extracted information concerning setting and methods (eg, country, context, study design, inclusion and exclusion criteria), participants (eg, total number of participants, missing participants, mean age, gender), outcome measures (method of assessing outcomes, duration), main findings (especially effectiveness summaries and analyses relating intervention content to effectiveness), intervention (eg, type of intervention, change targets in terms of cognitive, emotional, or physiological changes targeted and or assessed in process evaluations, mode of delivery, intervention content). Data extraction forms are available from the authors.

### Analyses

Each review was searched for descriptions of content of relevant self-directed weight loss interventions and for both statistical and narrative assessment of the relationship between intervention content and effectiveness. This information was extracted and is summarized for each review in Multimedia Appendix 2.

## Results

### Search Results

**Figure 1:**
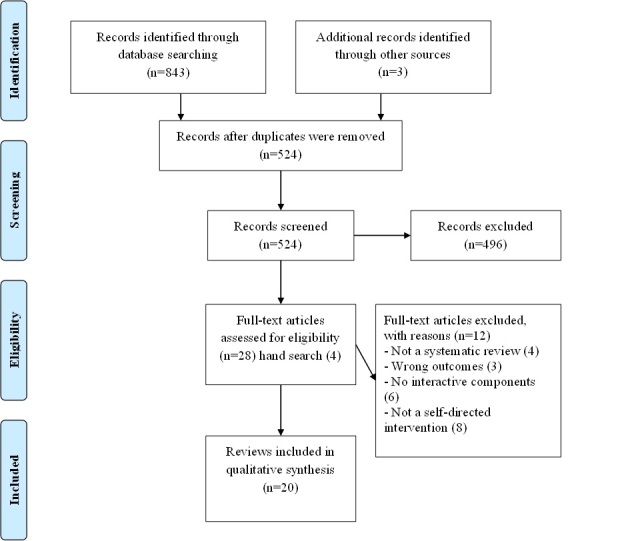
Study selection; PRISMA flow diagram.

In accordance with PRISMA (Preferred Reporting Items for Systematic Reviews and Meta-Analyses) guidelines, Figure 1 shows that our search strategy identified 524 articles after removal of duplicates. Title and abstract examination and reference-checking generated 32 potentially eligible articles of which 20 met our inclusion criteria. The number of participants included in studies within each review ranged from 298 to 12,417. Three of the selected reviews did not report total sample size.

### Review Characteristics

Included reviews were published between 2006 and 2012 and focused on weight loss in overweight or obese adults aged 20-79 years old. A summary description of each of the 20 reviews included is provided in Multimedia Appendix 2. The appendix describes review type, search period, inclusion criteria, OQAQ score, review aims, the number of primary intervention studies reviewed, the number of primary intervention studies meeting our inclusion criteria, the content of interventions meeting our inclusion criteria, and the overall results and conclusions.

Three of the selected reviews did not report total sample size across reviewed studies. Of the remaining, Enwald and Huotari [24] included the most participants (n=12,417) and Keller et al [25] the fewest (n=298). Most reviews focused on participants living in the community, although one review included home-based interventions for postpartum women [25].

All reviews, apart from one, summarized evaluation studies of weight loss or weight maintenance interventions. Three reviews excluded weight maintenance interventions [26-28]. Reviews included a variety of primary intervention evaluations, some relevant to our review and others not. For example, Kroeze et al reviewed the effectiveness of computer-tailored educational interventions evaluated in relation to a series of health-related outcomes, including smoking cessation, diabetes, and asthma management [29]. Consequently, only 2 of 31 primary evaluation studies included in this review were relevant to our research questions and so met our inclusion criteria. By contrast, all studies included in Weinstein met our inclusion criteria [30]. Thus for some reviews, we focus on conclusions based on a minority of the primary evaluations included in the review.

Only 4 reviews reported meta-analyses of weight-related outcomes [20,26,31,32]. The remaining 16 reviews reported narrative syntheses of primary studies (see Multimedia Appendix 2 for overview of reviews) [12,24,25,27,28-30,33-41].

### Review Quality

The overall methodological quality of included reviews was relatively poor. The average OQAQ score was 12.8. Individual review scores are shown in Multimedia Appendix 2. Only 5 reviews applied study quality assessment criteria to inform their analyses/interpretations [20,26,31,37,40], and most reviews did not assess the methodological quality of primary studies or consider potential reporting biases. Of the 20 included reviews, Loveman et al [37], Tuah et al [40], and Wieland et al [20] had the highest quality scores (of 18), and 4 others were high quality, scoring 17 [26,31], 15 [32], and 14 [33].

Apart from Neve et al [32], who did not use quality assessment criteria, all high-quality reviews examined the methodological quality of primary studies. Four reviews used the Cochrane Collaboration Risk of Bias Tool [20,26,31,40]. Harris et al also used the Effective Public Health Practice Project quality assessment index [31], and 2 reviews developed their own methodological quality assessments [33,37]. While all 7 high-quality reviews included primary evaluations of interventions for overweight or obese adults, Cole-Lewis and Kershaw [33] and Harris et al [31] also included interventions with adolescents, so their conclusions do not refer exclusively to adult populations.

### Weight Loss Effectiveness and Mode of Delivery

Across 20 reviews, we identified 99 primary evaluation studies that met our inclusion criteria. The interventions described in these studies employed a range of delivery formats including online programs, mobile phone applications, text messaging, email, electronic and print newsletters, telephone-based communication, print manuals, and booklets.

To explore findings, we grouped reviews according to the main delivery formats used by the interventions they considered. Nine reviews focused on Internet interventions. Three reviews evaluated interventions based on electronic devices such as mobile phones (referred to as “eHealth” interventions). Seven reviewed various multicomponent interventions, some of which were described as “home-based”, and one reviewed text-messaging interventions.

### Internet-Based Interventions

In a narrative review judged to be of medium quality, Weinstein included 8 evaluative studies (5 assessing weight loss and 3 assessing weight maintenance) [30]. All 8 met our inclusion criteria. The review included data from 418 overweight or obese participants aged 30-62 years with intervention durations of 6-12 months. Four of the five weight loss studies supported Weinstein’s conclusion that Internet-based interventions could provide an alternative to traditional interventions achieving weight loss of 1.7 kg (SD 2.7) to 2.2 kg (SD 2.6). The exception compared two self-delivered approaches. This study found that participants using a manual-based program lost substantially more weight than those using a tailored online dietary intervention. Findings were equivocal for the 3 weight maintenance interventions, and Weinstein called for further research.

Weinstein concluded that the content of Internet-based interventions is crucial to effectiveness and highlighted the potential importance of use of food records, sending weekly emails, and using telephone reminders. Weinstein called for further randomized trials on the use of Internet-based interventions for weight loss and weight maintenance [30].

Harvey-Berino et al randomized 250 participants to an Internet support group, minimal in-person support, or frequent in-person support group following a 6-month weight loss intervention involving interactive television. After 12 months, no differences were found. Interestingly, the Internet-based group experienced greater peer contact and were more likely to complete self-monitoring diaries but nonetheless had the highest attrition rate, suggesting that, for some participants, the intervention had diminishing appeal over time [42].

In a medium quality review, Kroeze et al report effectiveness of computer-tailored education on physical activity and dietary behaviors [29]. Only 2 of the 31 studies met our inclusion criteria because, although many assessed weight-related outcomes, most did not report weight lost. Results were inconclusive in relation to weight loss effectiveness.

In a narrative review judged to be low quality, Saperstein et al included 6 studies of online social support interventions that included feedback from a therapist, e-bulletin boards for peer support, and email communication with a counselor [28]. All 6 studies met our inclusion criteria. These interventions were effective with interventions achieving a weight loss range of 2.6-8.3kg, but only when specific change strategies were used. Information provision alone without feedback had no effect on weight outcomes. Saperstein et al concluded that “personalization through ongoing tailored information and feedback, either via email from a human counsellor or a computer-based program, was a critical component” (p. 4).

In another narrative review judged to be of medium quality, Turk et al included 40 studies that focused primarily on weight maintenance [41]. Only 8 of these studies met our inclusion criteria. Findings from 2 of these suggest that behavioral interventions with online chat sessions delivered via the Internet were as effective as an in-person behavioral therapy intervention [42,43]. Contrary to these findings, however, Harvey-Berino et al reported that an Internet chat group maintained significantly less weight than a minimal in-person and frequent in-person group (-5.7 kg [SD 5.9] vs -10.4 kg [SD 9.3] vs -10.4 kg [SD 6.3], respectively) [44]. Moreover, in Wing et al [45], an Internet chat room group was less successful in maintaining weight than in-person behavioral treatment (4.7 kg [SD 8.6] vs 2.5 kg [SD 6.7], respectively) [45]. These studies describe different interventions evaluated using different methods. The interventions evaluated in these studies vary greatly methodologically, which renders data synthesis impossible. Thus, findings in this review are equivocal, and the relative effectiveness of Internet versus face-to-face groups warrants further investigation.

Neve et al reported a high-quality meta-analytic review [32]. All 18 studies met our inclusion criteria. Random effects meta-analysis of 3 studies demonstrated a significant difference between an enhanced Web-based intervention (involving self-monitoring activities and individual email feedback) and an education-only Web-based intervention with less weight regained in the enhanced Web-based intervention group post intervention (weighted mean difference 2.24; 95% CI 1.27-3.21; *I^2^*=20.9%) [46-48]. Two weight maintenance studies combined in a meta-analysis also demonstrated less weight regain in participants involved in a Web-based intervention compared to a minimal intervention or usual care control group (weighted mean difference -0.30; CI -0.34 to -0.26; *I^2^=*0%) [49,50]. Although these meta-analyses supported the efficacy of Internet interventions, only 3 of the studies reported to achieve clinical weight loss of 5%. Neve et al were unable to draw generalizable conclusions due to the small numbers of comparable interventions [32].

In a narrative review judged to be of medium quality, Manzoni et al [38] updated Neve et al’s review [32] of 18 Internet-based studies, including 8 additional studies. All studies met our inclusion criteria and focused primarily on teenage women. Interventions lasted from 6 weeks to 2 years. Manzoni et al confirmed previous findings suggesting that Internet interventions including feedback are more effective than those providing information alone [38]. For example, Bennett et al randomized 101 participants to an Internet-based intervention including counseling sessions, behavior change goals, and self-monitoring compared to “usual care”. After 3 months, the intervention group lost 2.3 kg compared to a gain of 0.28 kg in the usual care group [51]. Overall, however, Manzoni et al judged available evidence to be inconclusive because of heterogeneity in duration and intensity of interventions, and variation in the type of feedback and social support tools used [38].

Manzoni et al also attempted to assess the cost-effectiveness of Internet-based interventions [38]. Two studies suggested that Internet-based interventions could be effective and less expensive than alternative interventions [52,53]. For example, Booth et al estimated that, over 12 weeks, Internet-based interventions could save up to US $155 compared to in-person interventions [52]. In addition, the authors reported further savings in travel time and travel costs after the 12-week period. However, only 2 studies provided cost-effectiveness data. Thus, further studies are needed to clarify how much less expensive Internet-based interventions could be when compared to standard weight loss interventions.

In a high-quality review, Reed et al identified 11 RCTs of Internet weight loss interventions, including email counseling and handheld, self-monitoring computer devices [26]. Seven of these studies, focusing on teenage women, met our inclusion criteria. Random effects meta-analyses of 6 of these studies found that adding a computer-based intervention to standard weight loss treatment significantly increased the amount of weight lost between 2 and 12 months (weighted mean difference −1.48 kg, 95% CI −2.52 to -0.43; *I* ^*2*^ *=*0%; *P=*.01) [54-58]. However, 4 of these studies were not primarily self-delivered interventions and so did not meet our inclusion criteria [54,55,57]. A meta-analysis of 5 exclusively self-directed computer-based interventions found that computer-based groups lost less weight than the standard treatment groups (using paper-based materials) (weighted mean difference 1.47 kg, 95% CI 0.13-2.81; *I^2^*=0%; *P<*.001) [59-62]. The authors concluded that Internet-based interventions were effective only when used in conjunction with a standard weight loss intervention, but the amount of weight lost (<1.5 kg) was too small to be clinically relevant for overweight and obese populations.

In a low-quality narrative review, Arem et al reviewed 9 RCTs, 8 of which met our inclusion criteria. These 8 studies reported modest weight loss ranging from 0.8-4.9 kg across studies [12]. For example, Hunter et al reported a 1.3 kg (SD 4.1) weight loss in 446 military participants who took part in an Internet-based intervention compared to a weight gain of 0.6 kg (SD 3.4) for those in a “usual care” group [63]. Rothert et al randomized 2862 participants to an online personalized information group and an online information-only group. After 6 months, the online personalized group lost significantly more weight (2.8 kg [SD 0.3] vs 1.1 kg [SD 0.4], respectively) [64]. Nonetheless, Arem et al [12] judged the data to be inconclusive due to the high attrition rates and variability/incompatibility of intervention methods. The most promising interventions identified in this review were in-person treatments followed by online weight maintenance, and only this combination achieved 5% weight loss. It is unclear, therefore, how effective Internet weight loss interventions are without personal contact/support.

Wieland et al reported a high-quality review examining the effectiveness of interactive computer-based interventions on weight loss and weight maintenance in obese or overweight adults [20]. All 18 studies reviewed met our inclusion criteria. These studies involved 4140 participants from the community, and 14 included weight loss as a primary outcome. For example, at 3 months, a meta-analysis of five weight loss trials found that computer-based intervention participants lost more weight than those in the minimal control group (eg, printed material or no treatment conditions; weighted mean difference -2.5 kg; 95% CI -3.4 to -1.6; *I* ^2^=44%) [51,56,65-67]. Four studies focused on weight maintenance (as opposed to initial weight loss) [43,45,49,50]. Participants using the computer-based interventions regained less weight than those in the minimal treatment or no treatment control at 6 months (mean difference -0.7 kg; 95% CI -1.2 to -0.2; two trials) [45,50] and at 12 months (mean difference -0.8 kg; 95% CI -1.4 to -0.2; three trials) [45,49,50]. One trial compared a computer-based intervention to an intensive in-person intervention (involving contact every 2 weeks over 12 months). Participants in the computer-based intervention regained more weight during the first 6 months (weighted mean difference 2.2 kg; 95% CI 0.3-4.1), and at 12 months lost less weight than the in-person group (weighted mean difference 4.7 kg; 95% CI 1.7-7.7) [43]. Overall, the authors of this high-quality meta-analytic review concluded that, compared to no intervention or minimal interventions, computer-based interventions are effective in prompting weight loss and in supporting maintenance of weight loss. However, computer-based interventions result in less weight loss and greater weight regain than in-person interventions. So, for example, while computer-based interventions may result in approximately 2.5 kg loss over 3 months, in-person interventions can achieve up to 10% of weight loss at up to 26 months [68].

### eHealth Interventions

Reviews used the term “eHealth” to refer to interventions delivered using electronic devices including smartphones and Internet-based computer interventions. Thus, the 3 reviews considered in this delivery category include primary evaluation studies that could also have been included in reviews of Internet intervention studies (as above).

In a narrative review judged to be of medium quality, Norman et al summarized 49 eHealth and Internet studies [39]. All studies targeted both dietary and physical activity behavior change, and interventions lasted 4-12 months. These studies were of variable quality, and only 12 met our criteria with 33 failing to include a weight-related outcome. Norman et al drew few conclusions but recommended that future studies focus on underlying mechanisms and change techniques that promote dietary and physical activity behavior change.

In a narrative review judged to be of medium quality, Enwald and Huotari evaluated electronic interventions for the prevention of obesity and its associated health problems [24]. Of the 23 included studies, 21 were RCTs and 2 employed quasi-experimental designs. However, only 5 studies met our inclusion criteria with 17 failing to include a weight-related outcome. Interventions included emails, use of websites, electronic feedback, CD-ROM, and newsletters and lasted between 1 and 12 months. Results showed that dietary interventions had a greater influence on weight than physical activity programs. Interestingly, tailoring was reported to be more effective when applied in the context of dietary interventions and less effective in physical activity programs.

By contrast, Harris et al conducted a high-quality meta-analytic review of 43 studies, of which 22 met our inclusion criteria; 21 did not include weight loss outcomes [31]. Across 40 adult studies participant ages ranged from 40-49 years. 27 of these studies used the Food Frequency Questionnaire, and others used a variety of outcomes. Interventions lasted between 1 and 6 months, and participants interacted with the intervention either daily or weekly for 10-45 minutes. Based on dietary behaviors outcomes, in particular intake of fruit, vegetable, fat, and fiber, this review found no evidence that eHealth was more effective or cost-effective than in-person interventions. Four self-delivered interventions reported mean weight [48,69-71], and three reported mean change in weight [49,50,58]. Random effects meta-analyses of both groups, that is, (1) the former four (weighted mean difference 0.6 kg; 95% CI -3.5 kg to 4.6 kg; *P=*.78) and (2) the latter three, found no evidence of intervention effect (weighted mean difference –0.07 kg; 95% CI –1.8 kg to 1.6 kg; *P=*.94). However, large heterogeneity of effect sizes casts some doubt on the applicability of these average results across intervention evaluations. In conclusion, however, this high-quality meta-analytic review found no evidence of effectiveness of self-delivered eHealth weight loss interventions in comparison with other approaches.

### Home-Based Print and Multicomponent Interventions

Three reviews summarized intervention evaluation studies, many of which were referred to as “home-based” [25,27,35]. These, together with interventions reviewed by 4 other reviews, typically included mailed instructions or advice on dietary and physical activity (eg, brochures, leaflets, health professional advice), self-monitored physical activity using electronic devices (eg, pedometers, accelerometers), promotion of diaries, and provision of various written materials—or a combination of the above [34,36,37,40].

In a narrative review judged to be low quality, Hemmingsson et al included 7 studies of physical activity, 2 of which met our inclusion criteria [35]. For example, Perri et al compared a “home-based”, individual walking intervention with an organized group-based walking intervention. At 12 months, participants in the individual intervention reported 20.8 minutes more walking per week than those in the group intervention. Those in the home-based group also lost more weight after 15 months (11.65 kg [SD 8.99] vs 7.01 kg [SD 8.23]) [72]. However, this was based only on a small sample of 49 obese women enrolled in a behavioral modification program. Further investigation of the potential of individual walking interventions is warranted.

In a narrative review of weight management interventions for postpartum women, judged to be low quality, Keller et al included 6 studies of which 3 met our inclusion criteria [25]. All 6 demonstrated significant changes in body composition with a reported weight loss range of 1.6-7.8 kg in 3 studies. For example, Leermakers et al found that the behavioral weight loss intervention group involving telephone contact and 16 written lessons on exercise, nutrition, and behavior change strategies lost more weight than the no treatment control group involving healthy eating and exercise informational brochures, after 6 months (7.8 kg vs 4.9 kg, respectively) [73].

In another narrative review judged to be low quality, Lemmens et al included 9 studies of interventions with adults of which 3 met our inclusion criteria [27]. The 9 interventions included home-based exercises, written materials, emails, and face-to-face sessions. Only one of these found a small but statistically significant weight loss difference of 1.6 kg.

In a high-quality narrative review, Loveman et al included 12 studies of multicomponent interventions that involved home-based weight loss schemes [37]. Of these, 10 met our inclusion criteria. Many of these studies reported small, average weight loss. However, variability in intervention duration, intensity, addition of subsequent weight maintenance intervention components, and length of follow-up prevented drawing of meaningful conclusions regarding common elements associated with effectiveness.

In a low-quality narrative review focused on weight gain prevention interventions, Lombard et al included 9 studies [36]. In general, low intensity multicomponent interventions combining physical activity, diet, and behavior change content were found to be effective for preventing weight gain. Weight loss range was 1-1.9kg for 7 studies matching our inclusion criteria. However, only 5 demonstrated significant findings. For example, in another study, Lombard et al examined an intervention consisting of four group-based behavior change sessions followed by text messages and monthly mail contact over a 1-year period. They found a difference of -1.01 kg (*P*=.03) of weight loss between the intervention and a control group (involving group-based education sessions) [74]. Overall, only a few studies assessed the effectiveness of interventions designed to prevent weight gain, and like other multicomponent reviews, intervention content varied across trials making it difficult to compare effect sizes and to generate robust conclusions.

Gordon et al reported a low-quality narrative review focusing on pharmaceutical and in-person weight loss treatment [34]. Two of the 10 included studies were primarily self-delivered and matched our inclusion criteria. For example, Ahrens et al compared an intervention incorporating personalized information sheets and tailored exercise advice to a reduced calorie diet group among 95 participants. After 6 months, no significant difference in weight loss was reported between groups [75]. Again variability across interventions made it difficult to draw conclusions about intervention components associated with effectiveness.

In a narrative review judged to be high quality, Tuah et al identified studies that applied the transtheoretical model (TTM) to weight loss, but only 2 of the 5 studies in this review were primarily self-delivered [40]. While these 2 studies reported a small change in weight, this was not sustained over 24 months. The authors concluded that “trials that used stages of change as an assessment and intervention framework, rather than just as a tool to assign and assess stage of change, reported minimal weight loss” (p. 18).

### Text Message Interventions

In a high-quality narrative review, Cole-Lewis and Kershaw summarized 12 studies of SMS text messaging (short message service, SMS) interventions promoting a range of health behaviors including smoking cessation, diabetes, and asthma management [33]. Only 2 of these studies met our inclusion criteria. Both reported effective text messaging interventions with a weight loss range of 2.9-4.5kg. For example, Haapala et al randomized 126 overweight adults aged 25-44 years to a text message or a no-contact control group. After 12 months, the intervention group lost more weight than the control group (4.5 kg/m^2^ vs 1.1 kg/m^2^, *P*=.006, respectively) [76]. Weight loss occurred mostly in the first 3 months when usage of the text message program was high, so the longer-term effects of text messaging were unclear.

### Change Mechanisms and Theoretical Frameworks

None of the 20 reviews drew conclusions regarding the usefulness of particular theories or mechanisms of change. However, some reviews did highlight theories underpinning intervention design.

Enwald and Huotari reported that the most commonly mentioned theory in the evaluation studies in their review was the transtheoretical model (TTM), which guided 14 of 23 studies [24]. Other theories used included the Elaboration Likelihood Model, the Precaution Adoption Model, the Theory of Reasoned Action, the Theory of Planned Behavior, Goal Setting Theory, and the Health Promotion Model. Enwald and Huotari did not relate the theoretical foundation of interventions to effectiveness.

Tuah et al identified two interventions applying TTM to weight loss, both of which resulted in small losses in weight that were not sustained over 24 months [40]. The authors reported that TTM-based interventions using feedback, self-monitoring, anthropometric measurements, and counseling resulted in significant effects on weight loss.

Harris et al considered use of theory and change mechanisms in relation to changes in fruit, vegetable, fiber, and fat intake [31]. Of the 13 effective interventions, they found only one study that employed theory to identify change mechanisms. Anderson et al reported that self-efficacy and outcome expectancies in relation to physical activity mediated greater consumption of fruit, fiber, vegetables, and fat [77]. However, no meditational analyses were conducted.

### Change Techniques and Delivery Formats

Only Wieland et al provided meta-analyses linking specific intervention components with effectiveness [20]. At 3 months, meta-analysis of 3 trials demonstrated that participants receiving Internet-based interventions supplemented with individualized feedback experienced greater weight loss than participants in an Internet-based intervention without individualized feedback (weighted mean difference -2.1 kg; 95% CI -2.9 to -1.4; *P*<.001) [46,48,78]. A similar effect was also found for participants using email counseling in 3 trials (weighted mean difference -2.3 kg; 95% CI -3.1 to -1.5; *P*<.001) [46,48,78] and automated feedback in one trial (weighted mean difference -1.8 kg; 95% CI -3.2 to -0.5; *P=*.009) [48] when compared to an Internet-intervention delivered alone.

At 3 months, non-directive email counseling did not induce significant weight loss in one trial (weighted mean difference –0.3 kg; 95% CI –2.2 to 1.7; *P*=.80) [78]. Similarly, no effect was found at 4 months for a group chat intervention delivered in conjunction with online self-monitoring (weighted mean difference 1.5 kg; 95% CI -0.7 to 3.7; *P=*.18). However, this was based on only one trial with no follow-up assessment beyond 4 months [79]. The authors concluded computer-based feedback delivered in conjunction with an Internet-based intervention enhances weight loss.

Brief descriptions of the intervention content identified in narrative reviews are included in Multimedia Appendix 2. The most commonly mentioned mechanism-based change “techniques” [17] across reviews were self-monitoring, feedback, and goal setting. Interventions including these change techniques were generally more effective than information only interventions. Reminders were used in a number of effective interventions as were self-efficacy enhancement techniques [18] and provision of counseling opportunities.

Manzoni et al noted that most effective interventions promoting weight loss and maintenance incorporated tailored feedback via email, e-counseling, food diaries, and self-monitoring of physical activity, diet, and weight [38]. However, in the absence of meditational analyses, it is unclear which combination of techniques and delivery formats enhanced effectiveness. Lombard et al observed that “self-monitoring of weight was a component of three [effective] interventions. Four interventions used self-monitoring of diet or physical activity, but the form or reason for monitoring was not always clear” (p. 2243) [36].

Neve et al explored intervention components within individual studies and concluded that social support, peer support contact, and online bulletin boards increased website usage but no meditational analyses were presented [32]. Similarly, Weinstein noted that effective interventions included “social” components such as e-counseling from a therapist and an online bulletin, but whether these components are directly linked to effectiveness requires further investigation [30].

Behavior change techniques were delivered by means of a variety of “delivery formats” including Internet sites, emails, text messaging, CDs, telephone calls, pedometers, paper questionnaires and diaries, manuals, pamphlets, booklets, brochures, and workbooks. However, reviews did not allow firm conclusions to be drawn regarding these delivery formats. Internet programs appear to be effective, especially in comparison with no intervention or minimal-contact interventions and have the capacity to enhance the effectiveness of in-person programs. Personal tailoring of programs may also enhance the effectiveness of self-directed weight loss interventions.

## Discussion

### Principal Findings

To our knowledge, this is the first systematic review of reviews to examine the effectiveness of self-directed weight loss interventions. Twenty reviews including 99 primary evaluations met our inclusion criteria. Only 7 of 20 reviews were high quality according to criteria specified by OQAQ but given the paucity of available evidence, we summarized evidence from all 20 reviews. The reviews identify a variety of potentially effective, self-directed weight loss interventions delivered by means of the Internet, mobile electronic devices, print media, and combinations of these delivery formats.

Three reviews focusing on Internet-based interventions and one focusing on eHealth interventions conducted meta-analyses to determine which intervention type, duration, and intensity were the most effective. Of these 4 meta-analytic reviews, the strongest evidence comes from Wieland et al, where all studies met our inclusion criteria [20]. For example, a meta-analysis of 5 trials demonstrated that self-delivered interactive computer-based programs were more effective than minimal interventions (eg, printed newsletters) or no treatment, for short-term weight loss and weight maintenance. This finding corresponds to that reported by Neve et al [32]. However, most trials included in these reviews did not examine weight outcomes beyond 1-year follow-up, so the impact of computer or Internet-based interventions on long-term weight loss is unclear.

Our first research question concerned the effectiveness of self-directed weight loss interventions. Reed et al [26] and Wieland et al [20] suggest that computer or Internet-based interventions are less effective than in-person treatment, but further trials are needed to clarify whether the greater weight loss observed following in-person treatments is replicable, clinically significant, and cost effective. Reed et al concluded that computer-based interventions delivered in conjunction with standard treatment enhance weight loss compared to standard treatment delivered alone. However, this meta-analysis included just 6 trials and the magnitude of weight lost advantage was small (<1.5 kg).

Overall, weight loss (kg) across all relevant studies reported in 7 reviews ranges from 0.8-7.8kg. Caution is advised in the interpretation of these figures as wide variations were found in intervention content and delivery. For example, most reviews did not report intervention intensity and frequency and of those that did, this varied from 1 week to 1 year. Five Internet-based studies within 2 reviews achieved a percentage weight loss of 5% [12,32], which has been used as a benchmark associated to health benefits [80,81]. However, most reviews did not report whether interventions achieve 5% weight loss. Therefore, it is unclear how many interventions achieved clinically significant weight loss.

Enhanced Web-based intervention involving self-monitoring activities and email feedback appear to be more effective than information-provision alone, but this conclusion was supported by only one meta-analysis including only 3 primary studies including 375 participants [32]. Currently no meta-analyses have been conducted examining text message, home-based print, and multicomponent delivery formats. Two primary studies reviewed by Cole-Lewis and Kershaw present promising findings in relation to short-term weight loss following text messaging interventions [33]. Narrative reviews of home-based print and multicomponent delivery formats are inconclusive, in part because of the heterogeneity of content found across these interventions.

Our second research aim was to investigate whether effectiveness is enhanced by inclusion of particular behavior change techniques. We found that underlying components within self-directed interventions that contribute to weight loss success were largely unexplored at both study and review level. No meta-analyses were available assessing associations between included techniques and weight loss. Reviewing single trials, Wieland et al observed that Internet-based interventions including individualized feedback or email counseling had been found to be more effective than Internet-based interventions that did not employ these techniques [20]. Conversely, interventions including non-directive email, group chat, and online self-monitoring had been found to be less effective than Internet-based interventions that did not employ these techniques.

Narrative reviews described the content of effective interventions and found that these tended to employ self-monitoring, feedback, and goal setting. However, these reviews did not examine whether such techniques were associated with enhanced weight loss or weight maintenance. No review presented evidence on dose-response data for included change techniques, so it remains unclear whether mere inclusion or frequency of technique use is important to efficacy.

Our third research aim was to investigate whether effectiveness is enhanced by using particular delivery formats. We found that definitive conclusions could not be drawn regarding the most effective delivery format for self-directed weight loss interventions. This may depend on target audience. A greater number of primary studies of Internet-based interventions are available, and these are found to be more effective than minimal interventions (such as provision of leaflets). Trials of other delivery formats, such as eHealth interventions and text messaging, suggest that such interventions can be effective. The advantages of all such interventions include personal tailoring of information, 24-hour availability, anonymity, online social support, and affordability. These characteristics imply that, when effective, such interventions are likely to be cost-effective. Unfortunately, many available evaluation studies are pilot or efficacy trials, rather than definitive trials, and few cost-effectiveness studies have been undertaken. Further evaluation studies using large samples with long-term weight loss follow up and cost-effectiveness analyses are needed.

### Strengths and Limitations

Our review identified a range of reviews including primary evaluation studies of self-directed interventions designed to reduce weight. Every effort was made to reduce bias in the search, selection of reviews, data extraction, and data analysis. This review provides an overview of what is currently known in this rapidly expanding research area.

Nonetheless, several challenges affecting our selection and interpretation of available evidence must be acknowledged. We relied on descriptions of interventions provided by reviewers, and these varied considerably in form and detail. Reviews also varied in their methods and in the quality of the review methodology employed with only 7 of 20 scoring highly on the OQAQ. In addition, the literature may well contain more primary evaluation studies that would meet our inclusion criteria than the 99 included in these 20 reviews. Furthermore, we found no reviews that used meta-regression [16] to examine relationships between intervention content and weight loss effectiveness, so suggestions rather than conclusions emerged in relation to our second and third research questions concerning the association between inclusion of particular behavior change techniques and use of particular modes of delivery and weight loss.

### What Further Research is Needed?

#### Comprehensive Review of Primary Evaluations

A comprehensive review of primary evaluations of self-directed weight loss intervention evaluation studies is needed. Such a review would capture studies beyond the 99 primary evaluations included in our 20 reviews. Such a review should compare interventions using similar delivery formats, taking account of the potentially varying content of comparison groups and relate techniques and materials to effectiveness.

Such a review should use a quality assessment tool to assess review methods. Some reviews identified here used the Cochrane Collaboration Risk of Bias Tool. This comprises questions divided into seven areas: generation of the allocation sequence, concealment of the allocation sequence, blinding, attrition and exclusions, other generic sources of bias, biases specific to the trial design (crossover or cluster randomized trials), and biases. Only one review used the Effective Public Health Practice Project Tool, designed for use in public health and including questions concerning eight specific areas: selection bias, study design, confounders, blinding, data collection methods, withdrawals and dropouts, intervention integrity, and analyses. Both tools are useful. The latter may be more appropriate when reviewing large scale population intervention evaluation studies.

#### Further Meta-Analyses Focusing on Intervention Components

As part of a comprehensive review of primary evaluation studies of self-directed weight loss interventions, meta-analyses focusing on high-quality evaluation studies could be used to identify common intervention components in studies segmented by delivery format. This would generate quantitative answers to questions such as “What content works best for website-based weight loss interventions?” and “What content works best for weight loss mobile phone applications?” [82]. This would extend the work of the 4 meta-analyses identified in our review [20,26,31,32] and provide clear answers to the second and third research questions we addressed. In addition, meta-regression, controlling for co-occurrence of change techniques across interventions, could clarify whether theory-based combinations of techniques enhance weight loss effectiveness [16]. Finally such a review should consider the varying content of comparison conditions (such as usual care or alternative interventions), as such control content has demonstrable effects on the observed efficacy of interventions [83,84].

#### Further High-Quality Primary Evaluations Comparing Different Modes of Delivery

Further high-quality primary evaluations that compare different modes of delivery for the same (or very similar) interventions within particular populations are needed. Such studies should be reported in accordance with CONSORT guidelines [85] and include lists and specification of behavior change techniques included in the intervention content design. Results could recommend whether particular approaches such as Internet site, text messages, or mixed methods home delivery are most likely to be effective. Such studies should clearly specify the nature of comparison groups, specifying what constitutes control conditions.

#### Further High-Quality Primary Evaluations Using Objective Measures of Weight Loss at Longer-Term Follow-Up

Further high-quality primary evaluations that use objective measures of weight loss should be used at longer-term follow-up to assess maintenance of weight loss. These should follow the recommendations above and include multiple weight assessments lasting over 1 or, ideally 2 or more years. Such evaluations, conducted to scale, could provide population effectiveness data rather than the efficacy data on initiation of weight loss provided by most current intervention evaluation studies.

### Conclusions

A systematic search identified 20 reviews including 99 primary evaluations of self-directed interventions designed to reduce weight. The evidence reviewed suggests that self-directed interventions can independently promote weight loss and can augment interventions involving personal contact. Some reviews identified techniques and delivery formats used in effective interventions, such as self-monitoring, feedback, self-efficacy enhancement, and social and peer support. However, it was not possible to infer which techniques or delivery modes are most strongly associated with increased weight loss for whom and in what contexts. Further primary evaluations of self-delivered weight loss interventions that clearly specify the behavior change techniques and materials employed are needed, especially with long-term follow-up. Further meta-analytic reviews focusing on weight loss intervention content and efficacy within delivery mode could provide better guidance for intervention designers and commissioners.

## Multimedia Appendix 1

Selection of the final 20 reviews applying inclusion criteria.

## Multimedia Appendix 2

Review characteristics.

## Abbreviations

BMI: body mass index
OQAQ: Overview Quality Assessment Questionnaire
RCT: randomized controlled trial
TTM: transtheoretical model
