# Supplementary material for: Self-Directed Interventions to Promote Weight Loss: A Systematic Review of Reviews
Source: J Med Internet Res. 2014 Feb 19;16(2):e58. doi: 10.2196/jmir.2857 (PMC3961624; doi:10.2196/jmir.2857)
Supplement: Supplementary file 1 [file jmir_v16i2e58_app1.pdf]

Multimedia Appendix 1

Selection of the final 20 reviews applying inclusion criteria

Author(s)	Date	Review Superseded	Systematic Review	Included interventions assessed Weight related outcome	Included interventions were self-directed	Reviewed interventions including interactive and self-regulatory components
1. Arem, H & Irwin, M	***2011***	***No***	***Yes***	***Yes***	***Yes***	***Yes***
2. Berkel, L. A & Carlos-Poston, W. S	2005	No	No	Yes	Yes	Yes
3. Cole-Lewis, H & Kershaw, T	***2010***	***No***	***Yes***	***Yes***	***Yes***	***Yes***
4. Enwald, H. P & Huotari, M. L	***2010***	***No***	***Yes***	***Yes***	***Yes***	***Yes***

5. Galani, C & Schneider, H	2007	No	Yes	Yes	No	No
6. Garaulet, M & Perez de Heredia, F	2009	No	No	Yes	Yes	Yes
7. Gordon, J & Watson, M	***2011***	***No***	***Yes***	***Yes***	***Yes***	***Yes***
8. Harris, J & Felix, L	***2011***	***No***	***Yes***	***Yes***	***Yes***	***Yes***
9. Hemmingsson, E & Page, A	***2001***	***No***	***Yes***	***Yes***	***Yes***	***Yes***
10. Holt, J & Warren, L	2006	No	No	Yes	No	Yes
11. Keller, C & Records, K	***2008***	***No***	***Yes***	***Yes***	***Yes***	***Yes***
12. Kroeze, W & Wekman, A	***2006***	***No***	***Yes***	***Yes***	***Yes***	***Yes***
13. Lemmens, V. E &	***2008***	***No***	***Yes***	***Yes***	***Yes***	***Yes***

Oenema, A						
14. Lombard, C. B & Deeks, A. A	***2009***	***No***	***Yes***	***Yes***	***Yes***	***Yes***
15. Loveman, E & Frampton, G. K	***2011***	***No***	***Yes***	***Yes***	***Yes***	***Yes***
16. Manzoni, G. M & Pagnini, F	***2011***	***No***	***Yes***	***Yes***	***Yes***	***Yes***
17. Maon, S & Edirippulige, S	2012	No	Yes	No	Yes	No
18. Miller, Y. D & Dunstan, D. W	2004	No	Yes	Yes	No	No
19. Neve & Morgan	***2010***	***Yes***	***Yes***	***Yes***	***Yes***	***Yes***
20. Norris, S. L & Zhang, X	2005	No	Yes	No	No	No
21. Norman, G. J & Zabinski, M. F	***2007***	***No***	***Yes***	***Yes***	***Yes***	***Yes***

22. Perry, K. J & Hickson, M	2011	No	Yes	Yes	No	Yes
23. Reed, V. A & Schifferdecker, K. E	***2011***	***No***	***Yes***	***Yes***	***Yes***	***Yes***
24. Saperstein, S. L & Atkinson, N. L	***2007***	***No***	***Yes***	***Yes***	***Yes***	***Yes***
25. Shaw, K. A & Gennat, H. C	2006	No	Yes	Yes	No	No
26. Shaw, K. A & O'Rourke, P	2005	No	Yes	Yes	No	Yes
27. Tuah, N. A & Amiel, C	***2011***	***No***	***Yes***	***Yes***	***Yes***	***Yes***
28. Turk, M. W & Yang, K	***2009***	***No***	***Yes***	***Yes***	***Yes***	***Yes***
29. Volpe, S. L	2006	No	Yes	No	No	No
30. Weinstein, P. K	***2006***	***No***	***Yes***	***Yes***	***Yes***	***Yes***

31. Wieland, L. S	***2012***	***No***	***Yes***	***Yes***	***Yes***	***Yes***
32. Winett, R. A & Tate, D. F	2005	No	No	Yes	Yes	Yes

Note. Selected reviews that meet inclusion criteria are highlighted in **Bold**.
