# Supplementary material for: Self-Directed Interventions to Promote Weight Loss: A Systematic Review of Reviews
Source: J Med Internet Res. 2014 Feb 19;16(2):e58. doi: 10.2196/jmir.2857 (PMC3961624; doi:10.2196/jmir.2857)
Supplement: Supplementary file 2 [file jmir_v16i2e58_app2.pdf]

Multimedia Appendix 2. Review characteristics.

First Author, Year	Type of review, intervention mode, search period & OQAQ score	Aim	Selection criteria	No of interventions, participants & sample type	Key outcomes	Reported intervention content and interactive components	Results and conclusions
Arem (2011)	Descriptive<br><br>Internet-based<br><br>2001 to Jan 2009<br><br>OQAQ=10	To examine the strengths and weaknesses of internet-based interventions	Inclusion: RCT; overweight or obese adults; weight loss or maintenance; web-based interventions	Interventions = 9<br><br>N= 4282<br><br>Sample = community; 79% female; predominately white	Weight loss and maintenance	Online tailoring; personalized emails on progress; self-monitoring diaries; individual feedback; structured weight loss lessons	8/9 relevant studies. No statistical pooling. High attrition rate. Effectiveness of internet interventions remains unclear
Cole-Lewis (2011)	Descriptive<br><br>Text messaging (mobile phones)<br><br>May and June 2009<br><br>OQAQ=14	To investigate the effectiveness of text messaging for disease prevention and management	Inclusion: Randomized or quasi-experimental controlled trials; text messaging for disease prevention or management; measures in health behavior, health outcomes, and/or clinical outcomes using pre post-tests; peer reviewed	Interventions = 12<br><br>N= 2365<br><br>Sample = predominately community; in 3 studies, women were greatly over-represented	Weight change. Most studies targeted other health behaviors	Text messaging; automated messaging; tailored messages	2/12 relevant studies. Weight loss of 2.9 and 4.5kg. Text messaging effective in weight loss and diabetes management

Enwald (2010)	Descriptive<br><br>E-health<br><br>Not restricted by publication date<br><br>OQAQ=12	Evaluation of second generation interventions to prevent obesity and its associated problems	Inclusion: RCT or Quasi-experimental; second generation technology; health behavior associated to nutrition; weight management and physical activity assessed using physiological or behavioral outcomes	Interventions = 23<br><br>N= 12417<br><br>Sample = unclear; gender NS; predominately white	Weight change	Personalized feedback as the tailoring mechanism used in most studies	5/23 relevant studies. Only two studies reporting weight loss of 4.1 and 5.3kg. Nutrition interventions more effective than PA promotion programs. Tailoring more effective in nutrition interventions than physical activity and weight management
Gordon (2011)	Descriptive<br><br>Community pharmacy-based<br><br>Jan 1999 to June 2009<br><br>OQAQ=10	To assess the cost-effectiveness and effectiveness of community pharmacy weight management interventions	Inclusion: Community pharmacy setting; weight management as primary focus	Interventions = 10<br><br>N= 2583<br><br>Sample = service users	Weight change	Self-monitoring and goal setting; questionnaires; diaries; information sheets	2/10 relevant studies. Did not synthesize quantitative data. Weight loss in one study at 3 and 6 months. Effectiveness remains unclear
Harris (2011)	Meta-analysis<br><br>E-health<br><br>Jan 1990 to Nov 2009<br><br>OQAQ=17	To assess the effectiveness and cost-effectiveness of e-health interventions for dietary behavior change and to determine the effective components and mechanisms	Inclusion: RCT; adolescents and adults; interactive and tailored software; dietary behaviors and clinical outcomes	Interventions = 43<br><br>N= Not stated<br><br>Sample = unclear;	Dietary behavior and clinical outcomes	Tailored software with goal setting and feedback components; personalized emails; self-monitoring diaries; personal digital assistant and automated telephone service; computer; CD-ROM; kiosks; minitel	22/43 relevant studies. Effectiveness and cost-effectiveness of e-health interventions inconclusive
Hemmingsson (2001)	Descriptive<br><br>Internet-based<br><br>1966 to 2001	A summary of physical activity behavior change adherence influences	Inclusion: Overweight and obese adults; contain PA behavior change adherence information	Interventions = 7<br><br>N= 651<br><br>Sample = unclear	Physical activity levels and weight/fat loss	Insufficient description of intervention content; peer support; telephone social	2/7 relevant studies. Did not synthesize quantitative data. Various factors influencing PA behavior change.

	OQAQ=8					support and logins described in two relevant studies	Evidence not conclusive and warrants further research
Keller (2008)	Descriptive<br><br>Home/gym based exercises for postpartum women<br><br>May 2007<br><br>OQAQ=9	A description of intervention studies for guiding weight management interventions	Inclusion: Weight loss or body composition outcome; PA interventions; postpartum women; RCT	Interventions = 6<br><br>N= 298<br><br>Sample = community; 100% female; predominately white	Weight and fat loss	Food and activity diaries for self-monitoring and goal setting; Harris-benedict equation; target heart rate percentage; motivational interviewing; informational brochure; written lessons; telephone contact	3/6 relevant studies. Weight loss range of 1.6 to 7.8kg. Significant changes in body composition in women found in relevant studies. No significant changes in weight loss. Effectiveness remain unclear
Kroeze (2006)	Descriptive<br><br>Computer-based<br><br>1965 to Sep 2004<br><br>OQAQ=12	A review of the efficacy of nutrition education and expert-driven computer-tailored physical activity interventions	Inclusion: Computer tailored interventions targeting PA or diet; RCT with pre and post-test; adults; studies in English; delivered in a “nonpersonal” way without person-to-person counselling	Interventions = 31<br><br>N= 84-1,317<br><br>Sample = unclear	Change in health behavior (PA and diet) and weight loss	Tailored and interactive feedback; computer-tailored letters; pamphlets; brochures; multimedia (text and video); computer-tailored print materials	2/31 relevant studies. Did not synthesize quantitative data. Evidence for computer-tailored interventions is strong. However, small effect size in most studies. Follow up periods only up to 6 months
Lemmens (2008)	Descriptive<br><br>Home-based; group; face to face; written materials and emails<br><br>1998 to 2008<br><br>OQAQ=9	To examine the efficacy of obesity prevention interventions and to identify the effective components for intervention development	Inclusion: Dietary intake; PA or both; overweight individuals; RCT; non-RCT or interrupted time series designs; minimum 3 months follow up	Interventions = 9<br><br>N= not stated<br><br>Sample = community; predominately white; 20-79 years old	BMI and weight loss	Emailed exercise and dietary programmes; self-monitoring	3/9 relevant studies. Only one study targeting diet and PA demonstrated significant weight loss of 1.6kg. Inconclusive findings for the effectiveness of home-based interventions
Lombard (2009)	Descriptive<br><br>Includes a range of	To summarize weight gain prevention	Inclusion: Adults; weight gain prevention;	Interventions = 8<br><br>N= 2282	Weight loss	Self-monitoring; tailored advice;	7/9 relevant studies. Small differences in weight lost between

	delivery including face to face; groups; mail; newsletters and phone<br><br>1998 to 2008<br><br>OQAQ=10	interventions to identify the key elements contributing to intervention success	comparison group; duration of 3 months or over; follow-up < 3 months; family studies targeting adults	Sample = unclear		social support; problem solving; goal setting; stress and time management; relapse prevention; self-weighing; cognitive and behavior change strategies; personalized contact; newsletters; email and phone contact; incentives; booklet; pedometer	treatment groups. Weight loss range of 1 to 1.9 kg. Five studies reported significant weight loss. Few trials on weight gain prevention.<br>Low intensity multicomponent interventions identified as most effective
Loveman (2011)	Descriptive<br><br>Multicomponent<br><br>No restrictions (from inception to 2009)<br><br>OQAQ=18	To evaluate the long-term cost and clinical effectiveness of multicomponent weight management interventions for weight loss and maintenance	Inclusion: RCT; adults (≥ 18 years); overweight or obese; structured multicomponent weight management programmes; diet, PA or both; lifestyle behavior change strategy	Interventions = 12<br><br>N= 59-1191<br><br>Sample = predominately white females. However, some studies did not mention participant characteristics	Weight loss	Self-monitoring; goal setting; individual based problem solving; incentives; stages of change; rewards; stress management; modelling; assertiveness training; cognitive behavioral strategies; relapse prevention model; pocket diaries; lotteries to encourage attendance; questionnaires	10/12 relevant studies. Did not synthesize quantitative data. Weight lost in overweight and obese adults, but not clinically significant. Effectiveness remains unclear. No UK RCT studies in review

Manzoni (2011)	Descriptive<br><br>Internet-based<br><br>18 studies from previous review (Neve et al. 2010) and 2008 to 2011 for own review<br><br>OQAQ=13	To examine the effectiveness of web-based interventions for weight loss and maintenance	Inclusion: RCT; publication in a peer reviewed journal; overweight or obese adults; internet-based; weight loss or maintenance outcomes	Interventions = 26<br><br>N= 8324<br><br>Sample = community; 77% female	Weight loss and maintenance	Individual e-mail feedback; self-monitoring of weight and diet; exercise diaries	25/25 relevant studies. Heterogeneity found in most studies including duration of intervention, type of feedback, and social support tools. Findings inconclusive
Neve (2010)	Meta-analysis<br><br>Web-based<br><br>1995 to 2010<br><br>OQAQ=15	To examine the effectiveness of internet-based interventions for weight loss and weight maintenance and to identify the underlying components influencing weight control	Inclusion: RCT with one web-based intervention study arm; targets weight loss or maintenance; dietary or PA behavior change; 18 years or over; overweight or obese	Interventions = 20<br><br>N= 5700<br><br>Sample = 77% female (3.7% unclear)	Percentage change in body weight	Behavioral therapy; individually tailored web-based programme; email feedback; motivational interviewing; telephone follow-up; human email counselling	18/18 relevant studies. Three of six studies reported 5% or greater weight lost. Heterogeneity between studies. Effectiveness remains unclear. Require studies providing detailed descriptions of intervention content for meta-analyses
Norman (2007)	Descriptive<br><br>E-health technology<br><br>No restrictions (from inception to 2007)<br><br>OQAQ=13	To provide a descriptive evaluation of interactive second generation e-health interventions to promote weight loss	Inclusion: RCT and quasi-experimental; published in scientific journal; PA; dietary behavior or a combination of both; baseline and follow-up measures; intervention feasibility or effectiveness; e-health	Interventions = 47<br><br>N=<br>PA = 28-655;<br>diet = 72-1578;<br>combination = 35-2121<br><br>Sample = unclear but female participants<br>PA = 64-100%;<br>diet = 70-100%;<br>combination = 49-100%; predominately white females	Weight loss and BMI	Motivational tips; educational information; internet tailored newsletters; computerized assessment and feedback; electronic self-monitoring forms; diet analysis; chat room; bulletin board; therapist-led internet video sessions; online meetings and support; buddy program; animated fitness instructor; 24-	12/49 relevant studies. Did not synthesize quantitative data. Small to medium effect sizes reported across e-health studies. Findings inconclusive. Require further studies to understand underlying mechanisms promoting dietary and physical activity behavior change

						hour help desk; e-mail reminders; goal setting; interactive multi-media (IMM); CD-ROM; interactive phone technology	
Reed (2011)	Meta-analysis<br><br>Internet-based<br><br>No restriction date reported<br><br>OQAQ=17	To investigate the influence of computer technology on weight loss interventions	Inclusion: RCT; computer-based education or support; non-computer-based comparison group; overweight or obese adults; weight or BMI outcomes	Interventions = 13<br><br>N= 1866<br><br>Sample = means age = 49; 67% female; predominately white	Weight loss and BMI	Paper diary; booklet; workbook; group therapy; in-person sessions; paper-based and phone counselling	7/11 relevant studies. Computer-based interventions effective in weight loss but only when used in addition to standard weight loss programs. Sustaining weight loss over time remains unclear
Saperstein (2007)	Descriptive<br><br>Internet-based<br><br>Not reported<br><br>OQAQ=9	To investigate the efficacy of the internet as a mode of delivery to facilitate weight loss.	Inclusion: RCT; internet-based weight lost programs; overweight or obese adults	Interventions = 6<br><br>N= NS<br><br>Sample = unclear	Weight and waist circumference	Online logins; food diaries; online programs targeting dietary and PA changes, through cognitive and behavior strategies; personalized feedback and support	6/6 relevant studies. Did not synthesize quantitative data. Internet-based tools could be effective for weight loss. Particularly, structured, personalized programs that include cognitive behavioral strategies
Tuah (2011)	Descriptive<br><br>Range of methods incorporating the Transtheoretical Model and Stages of Change TTM SOC<br><br>No restriction (from inception to 2011)	To examine the effectiveness of PA and dietary programs that use the transtheoretical model (TTM) to facilitate weight loss	Inclusion: RCT; 18 years or over; overweight or obese; healthy adults; co-morbidities including diabetes, heart diseases and hypertension; use of TTM SOC model; tailored lifestyle modification strategies	Interventions = 5<br><br>N= 3910<br><br>Sample = age range from 25-75, predominately white	Weight loss	TTM SOC; self-monitoring; feedback; personalized assessment report quarterly telephone; newsletters; manuals; pedometers; face to face counselling	2/5 relevant studies. Interventions utilising TTM SOC induced small weight change, but weight lost unsustainable over time. Heterogeneity across outcomes and interventions with small to medium samples. TTM SOC ineffective for weight loss

	OQAQ=18						
Turk (2009)	Descriptive<br><br>Clinical, therapy and self-delivered modality<br><br>1984 to 2007<br><br>OQAQ=11	To summarize the findings of RCT interventions that assess strategies for maintenance of weight and the viability of these programs	Inclusion: RCT; weight maintenance; adult population; English language; true experimental design; randomly assigned at maintenance	Interventions = 40<br><br>N= unclear<br><br>Sample = predominately white	Weight maintenance	Self-monitoring; self-weighing; chatroom; weekly weights online; 3-day food diaries; healthy diet educational materials; pedometers	8/40 relevant studies. Mixed results. No difference in weight maintenance when compared to in-person interventions in two relevant studies
Weinstein (2006)	Descriptive<br><br>Internet-based<br><br>1995 to 2006<br><br>OQAQ=12	To investigate the efficacy of weight loss programs delivered via the internet	Inclusion: RCT; publication in peer reviewed journal; overweight or obese adults; internet-based interventions; weight loss	Interventions = 8<br><br>N= 418<br><br>Sample = age range from 30-62; 55% female; predominately white	Weight loss and maintenance	Weekly feedback; email; bulletin board support; email from therapist; e-counselling; food diaries; tailored health information and phone support	8/8 relevant studies. No quantitative synthesis. Weight loss range of 0.8 to 2.2kg across 5 studies. Internet weight-loss effectiveness similar to standard weight loss programs
Wieland (2012)	Meta-analysis<br><br>Computer-based<br><br>From inception to 2011<br><br>OQAQ=18	To examine the effectiveness of interactive computer-based interventions for weight loss or weight maintenance	Inclusion: RCT and quasi-randomized trials; computer-based interventions; interactive programs; overweight or obese adults	Interventions = 18<br><br>N = 4140<br><br>Sample = predominately white	Weight loss and maintenance	Interactive; weekly online chats; weekly email feedback; electronic bulletin board; e-mailed individualized feedback; e-mail counselling; online self-monitoring; online education or online behavior therapy; message boards	18/18 relevant studies. Interactive computer-based interventions are more effective than minimal (usual care, pamphlets) or no interventions
